# Increasing connections among temporal invariability, resistance and resilience of alpine grasslands on the Tibetan Plateau

**DOI:** 10.3389/fpls.2022.1026731

**Published:** 2022-11-09

**Authors:** Yuting Yang, Yi Sun, Ben Niu, Yunfei Feng, Fusong Han, Meng Li

**Affiliations:** ^1^ School of Geographic Sciences, Nantong University, Nantong, China; ^2^ Lhasa National Ecological Research Station, Key Laboratory of Ecosystem Network Observation and Modelling, Institute of Geographic Sciences and Natural Resources Research, Chinese Academy of Sciences, Beijing, China; ^3^ Department of Resource Management, Tangshan Normal University, Tangshan, China

**Keywords:** alpine grassland, grazing, climate change, stability, dimensionality

## Abstract

Ecological stability contains multiple components, such as temporal invariability, resistance and resilience. Understanding the response of stability components to perturbations is beneficial for optimizing the management of biodiversity and ecosystem functioning. Although previous studies have investigated the effects of multiple perturbations on each stability component, few studies simultaneously measure the multiple stability components and their relationships. Alpine grasslands on the Tibetan Plateau are exposed to co-occurring perturbations, including climate change and human activities. Here, we quantified three stability components (temporal invariability, resistance, and resilience) of alpine grasslands on the Tibetan Plateau during periods of high (2000-2008) and low (2009-2017) human activity intensity, respectively. We focused on the effects of climate variables (temperature, precipitation, radiation) and human activities (grazing intensity) on covariation among stability components. The results show that (1) for periods of high and low human activity, temporal invariability was positively correlated with resistance and resilience, while resistance was independent of resilience; (2) the dimensionality of alpine grasslands decreased by almost 10%, from 0.61 in the first period to 0.55 in the second period, suggesting the increasing connections among temporal invariability, resistance and resilience of alpine grasslands; and (3) temperature but not grazing intensity dominated the changes in the dimensionality of stability. These findings improve our understanding of multi-dimensional stability and highlight the importance of climate variability on alpine grassland stability on the Tibetan Plateau.

## Introduction

Climate change and human activity are increasingly affecting the ecological processes of terrestrial ecosystems (IPCC, 2014; Yang et al., 2017b). Ecosystem stability becomes the core of understanding the ecosystems’ ability to maintain or recover their structure and functions under global change (Bai et al., 2004; Pennekamp et al., 2018; Radchuk et al., 2019). In arid and semiarid regions, biodiversity loss and productivity reduction due to the unsustainable land use and drastic climate change have weakened grassland stability strongly (Hooper et al., 2012; Haddad et al., 2015). There is a consensus that intense stressors may transit grassland to a catastrophic state until grassland stability is completely lost (Qin et al., 2019; Song et al., 2020). At the plot level, mounting empirical studies have investigated the underlying mechanisms by which grassland ecosystems maintain relatively stable levels to avoid catastrophic transition at the plot scale (Grman et al., 2010; Ma et al., 2017; Saruul et al., 2019). However, although the impacts of climate change and human activities on ecosystem functions have been studied globally, little research has assessed the ecosystem stability in response to these perturbations in a large scale.

The satellite-derived vegetation indices, such as normalized difference vegetation index (NDVI) and Enhanced Vegetation Index (EVI), are directly associated with vegetation activity and have been widely used to investigate the responses of ecosystem productivity to perturbation (Del Grosso et al., 2018; Lu et al., 2019). From NDVI or EVI anomalies in time series, previous studies have successfully extracted different large-scale ecosystem stability components, such as temporal invariability, resistance and resilience (De Keersmaecker et al., 2015; Li et al., 2020; White et al., 2020). Temporal invariability measures the fluctuations of community biomass production over time, resistance quantifies ecosystems’ ability to absorb perturbations, and resilience reflects the capacity of a disturbed ecosystem recovering to its original state (Pimm, 1984; White et al., 2020). These stability components are usually correlated each other (Pennekamp et al., 2018; Kefi et al., 2019). For example, resistance is expected to correlate positively with temporal invariability but negatively with resilience (Li et al., 2020; Chen et al., 2021). Donohue et al. (2013) argued that correlations among stability components construct the dimensionality of ecosystem stability which contains abundant information for predicting the response of ecosystems to environmental change. Strong correlations among stability components can shape a low effective dimensionality of stability, implying similar processes and maintenance mechanisms underlie ecosystem stability components. Therefore, the monitoring schemes can be optimized by quantifying only a few stability components when the dimensionality of stability is low (Radchuk et al., 2019). In contrast, if these stability components are uncorrelated, ecosystem stability will shape a high dimensionality, suggesting that ecosystems may respond to perturbations in many compensatory ways. In this case, policymakers should measure all stability components to manage natural systems (Donohue et al., 2013; Radchuk et al., 2019).

Theoretically, ecosystems generally keep a relatively stable level in fluctuating environmental conditions. However, large or high-frequency perturbations tend to prompt ecosystems in a highly variable state (Pimm, 1984). On the one hand, rapid climate change, e.g., global warming accompanied by precipitation patterns change, can shift community composition and influence species interactions, thus changing ecosystem stability (Crossman et al., 2012; Ma et al., 2017; Antão et al., 2022). On the other hand, anthropogenic perturbations that affect species diversity and their interactions may therefore decrease the ability of an ecosystem to adapt to or resist environmental changes (Tilman and Lehman, 2001; Venter et al., 2016). For example, overgrazing can typically shift dominant species and change functional traits of persistent species, further reducing biomass stability (Grice et al., 2008; Wu et al., 2013). The intermediate disturbance hypothesis supported that moderate grazing can reorganize the spatial pattern of plants and nutrients to optimize utilization of the limiting resources, such as water and light, ultimately enhancing plants’ ability to resist perturbations (Song et al., 2020). Therefore, investigating the coupling effects of climate change and human activity on ecosystem stability help understand how ecosystems respond to perturbations.

Alpine grassland on the Tibetan Plateau is one of the fragile ecosystems on Earth and is crucial for maintaining the social economy’s sustainable development and the plateau’s ecological balance (Yao et al., 2012; Yang et al., 2017a). Over the last several decades, this plateau has experienced rapid and general warming and a tremendous increase in livestock numbers (Chen et al., 2013; Cao et al., 2019). These perturbations have caused extensive grassland degradation across the plateau (Harris, 2010). Since 2000, various ecological protection policies have been continuously renewed and implemented to protect and restore degraded grasslands on the plateau. After 2009, human activity intensity on alpine grasslands declined significantly, gradually shifting the ecological environment in a positive direction (Li et al., 2021b). Although many studies have assessed the response of grassland productivity to these policies and climate change, how grassland stability responds to these perturbations is still unknown. In this study, based on the inter-annual dynamic of grassland NDVI and its corresponding climate variables, i.e., temperature, precipitation and radiation, we first calculated three stability components of temporal invariability, resistance, and resilience at the regional scale. Then, we quantified the dimensionality of stability based on correlations among the three stability components. Finally, we compared the difference in stability components between the two periods to clarify the relative contribution of climate variables (refers to temperature, precipitation and radiation) and human activities (refers to grazing intensity) to grassland stability changes. This study aims (a) to investigate the changes in grassland stability components and their relationships, and (b) to explore the primary factor in regulating grassland stability on the Tibetan Plateau.

## Materials and methods

### Study area

**Figure 1:**
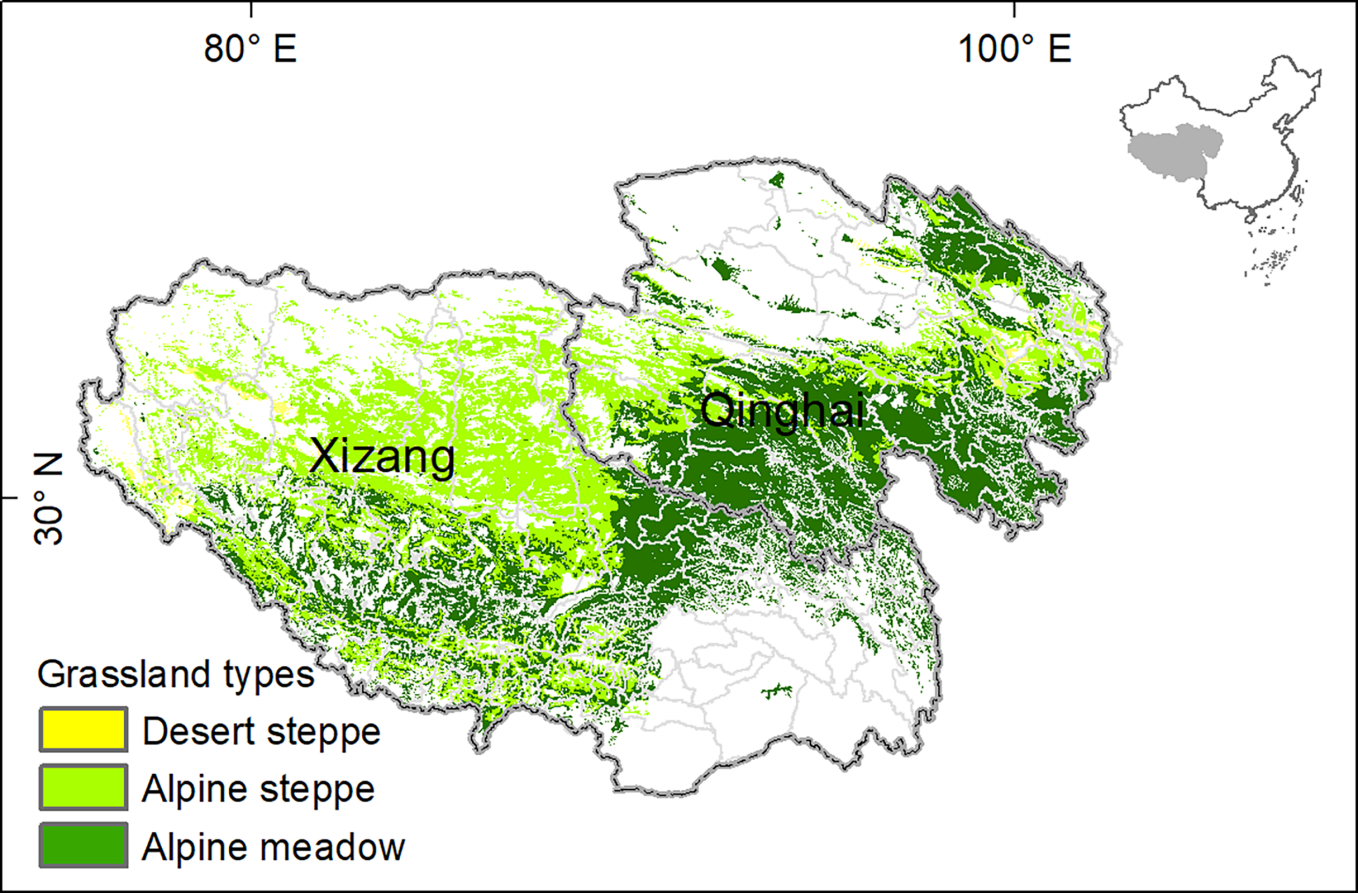
Spatial distribution of grasslands on the Tibetan Plateau.

The Tibetan Plateau in China starts from the foot of the Himalayas in the south, and reaches the foot of the Kunlun Mountains and the Qilian Mountains in the north. This plateau occupies around one-quarter of surface areas in China, with an average altitude of 4,000 m above sea level. Known as the Asian Water Tower, this region is the headwaters of the most important Asian rivers, such as the Yellow River, Yangtze River, Mekong River and Brahmaputra River, and thus plays an essential role in maintaining biodiversity and biogeochemical cycles of its surrounding countries (Yao et al., 2012). Alpine grassland, the most distributed vegetation type, is the critical animal husbandry base and ecological barrier of the Tibetan Plateau (Long et al., 1999). Xizang Autonomous Region and Qinghai province are the major section of the Tibetan Plateau, separated by the Tanggula Mountains (**Figure 1**).

### Data and processing

We downloaded Normalized Difference Vegetation Index (NDVI) from NASA’s moderateresolution imaging spectroradiometer (MODIS) (MOD13A3) from 2000 to 2017. This dataset was produced at a spatial resolution of 1,000 m and a time step of one month. The noises in time-series NDVI data, such as atmospheric effects and cloud contamination, were calibrated based on the Savitzky–Golay method (Chen et al., 2004). Previous studies suggest that the low quality of NDVI data for sparsely vegetated lands on the Tibetan Plateau may affect further analyses of ecosystem processes (Zu et al., 2018; Hua et al., 2022a). Thus, any pixel with annual NDVI lower than 0.1 was eliminated to reduce the potential effects of noisy data. The daily meteorological data, including temperature, precipitation and sunshine hours, were collected from the National Meteorological Information Center of China Meteorological Administration. We first aggregated daily meteorological data to monthly level to match the temporal resolution of the NDVI data. Then, we used ANUSPLIN 4.3 to interpolate monthly climatic records into raster surfaces with a 1,000 m resolution (Hutchinson, 2004). Solar radiation was calculated based on sunshine hours with the FAO procedure (Allen et al., 1998). The quality of grid climatic surfaces has been demonstrated by the high correlations to field observation records (Chen et al., 2014; Tao et al., 2015). Information on the distribution of grasslands was obtained from the vegetation atlas of China with a scale of 1:1000 000 (Editorial Committee for Vegetation Map of China, 2001). Livestock records for each county on the Tibetan Plateau, including sheep, goat, yak, donkey, and horse, were downloaded from statistical yearbooks (https://kns.cnki.net/kns8?dbcode=CYFD).

### Grazing intensity

In this study, we measured the grazing intensity (GI) for each county on the Tibetan Plateau. We first converted livestock numbers to standard sheep units (SHU) to ensure comparability between different counties. As suggested by previous studies, one sheep or one goat equals one SHU, and one yak, one donkey, or one horse equals four SHUs (Li et al., 2021a; Yu et al., 2021). Then, we extracted the area of available natural grassland (ha) in each county from the information of grassland distribution. Finally, we calcluated the GI as the ratio of livestock number to natural grassland areas.

### Calculation of temporal invariability, resistance and resilience

In this study, we calculated three stability components, including temporal invariability, resistance, and resilience, based on the temporal NDVI and climatic variables. Prior to analysis, all-time series of NDVI and the three climate variables were detrended using a linear regression model. Temporal invariability was computed as the ratio of long-term mean of a NDVI value to its standard deviation (Deléglise et al., 2011; Ma et al., 2017). Resistance and resilience were quantified through the relationships between NDVI and climate variables, as did in previous studies (De Keersmaecker et al., 2015; Seddon et al., 2016; Li et al., 2018). A linear autoregressive model was used to build the relationship between NDVI anomaly and climate anomaly (temperature, precipitation and solar radiation). In this model, all variables were transformed to *z*-score anomalies using variables’ means and standard deviations. The coefficients of the linear model were used to measure ecosystem resistance and resilience.


$${\text{NDVI}}_{\text{t}}=\text{α}\times {\text{T}}_{\text{t}}+\text{β}\times {\text{P}}_{\text{t}}+\text{γ}\times {\text{R}}_{\text{t}}+\text{δ}\times {\text{NDVI}}_{t-1}+\text{ϵ}$$

where NDVI_t_ is the NDVI anomaly at time t; T_t_, P_t_ and R_t_ are the corresponding temperature, precipitation and radiation anomaly, respectively; NDVI_t-1_ is the NDVI anomaly at time t-1, and ϵ is the residual error. α, β and γ are temperature resistance metric, drought resistance metric and radiation resistance metric, which can be used to calculate the ecosystem’s resistance to climate change. δ indicates the similarity between the current state and the previous state, which can be considered a resilience metric. A higher absolute δ value represents lower resilience (De Keersmaecker et al., 2015; Li et al., 2018). More detailed information for calculating ecosystem resistance and resilience can be found in Li et al. (2018). Finally, temporal invariability, resistance and resilience of grassland ecosystems were normalized between 0 and 100 using the minimum and maximum values.

### Correlations among invariability, resistance and resilience

We used the space-for-time substitution to calculate the correlations among stability components. Firstly, we coarsened gridding cells of alpine grassland to the administration unit based on the boundary of 122 counties (districts) on the Tibetan Plateau. Then, we divided grassland cells in each county(district) into different grassland types. We finally generated 206 spatial datasets to analyze the correlations among invariability, resistance and resilience.

For each spatial dataset, we calculated and tested Pearson correlations between pairs of the three stability variables and constructed covariance matrices with these Pearson correlations to measure the dimensionality of stability. The dimensionality of stability represents the strength of the relationship among various stability components (Donohue et al., 2013). The magnitude of dimensionality is equal to the volume of the ellipsoid (V), which is constructed by covariance matrices (Donohue et al., 2013):


$$a_{i}=\lambda_{i}$$

$$\text{V=}\frac{{\text{π}}^{\text{n}/2}}{\text{Γ(}\frac{\text{n}}{2\;}+1)}\prod_{\text{i}=1}^{\text{n}}\left(\lambda_{i}^{0.5}\right)$$

where *a_i_* is the semi-axis length of the ellipsoid, V is the volume of the ellipsoid, n is the dimensionality of the covariance matrix, *λ_i_* is the i^th^ eigenvalue of the covariance matrix. For each covariance matrix, semi-axis lengths are normalized by dividing the maximum length among all the semi-axis lengths so that the maximum standardized length equaled 1. Thus, proportional volume can be normalized by dividing the actual ellipsoid volume by the theoretical maximum between 0 (low dimensionality, a ‘cigar’-like shape of ellipsoids) and 1 (high dimensionality, a perfect sphere). If the dimensionality is high, the stability components are independent (the relationship among various stability components is poor), and vice versa.

### Statistical analysis

Pearson correlation analysis was performed to examine the pairwise correlations among stability components. One-way analysis of variance (ANOVA) was used to examine the differences in stability between different types. We employed random forest algorithm with the *randomForest* package in R 4.1.2 to evaluate the relative contribution of climate variables and grazing intensity to variation in grassland stability. Random forest algorithm is a high accuracy and robust machine learning algorithm, which is suitable for processing high-dimensional and -correlated datasets (Tin Kam, 1998; Breiman, 2001; Xia et al., 2018). In the random forest model, we selected the trends in MAT, AP, AR, and GI (Trend_MAT_, Trend_AP_, Trend_AR_ and Trend_GI_, respectively) as predictors because they are closely correlated with ecosystem stability and are widely used in determining the driving mechanisms of vegetation change (Wessels et al., 2004; Mariano et al., 2018; Li et al., 2021b). The mean square error (%IncMSE) of each predictor in the random forest model represents variable importance, and higher %IncMSE values indicate higher variable importance.

## Results

### Spatial differences in invariability, resistance and resilience

**Figure 2:**
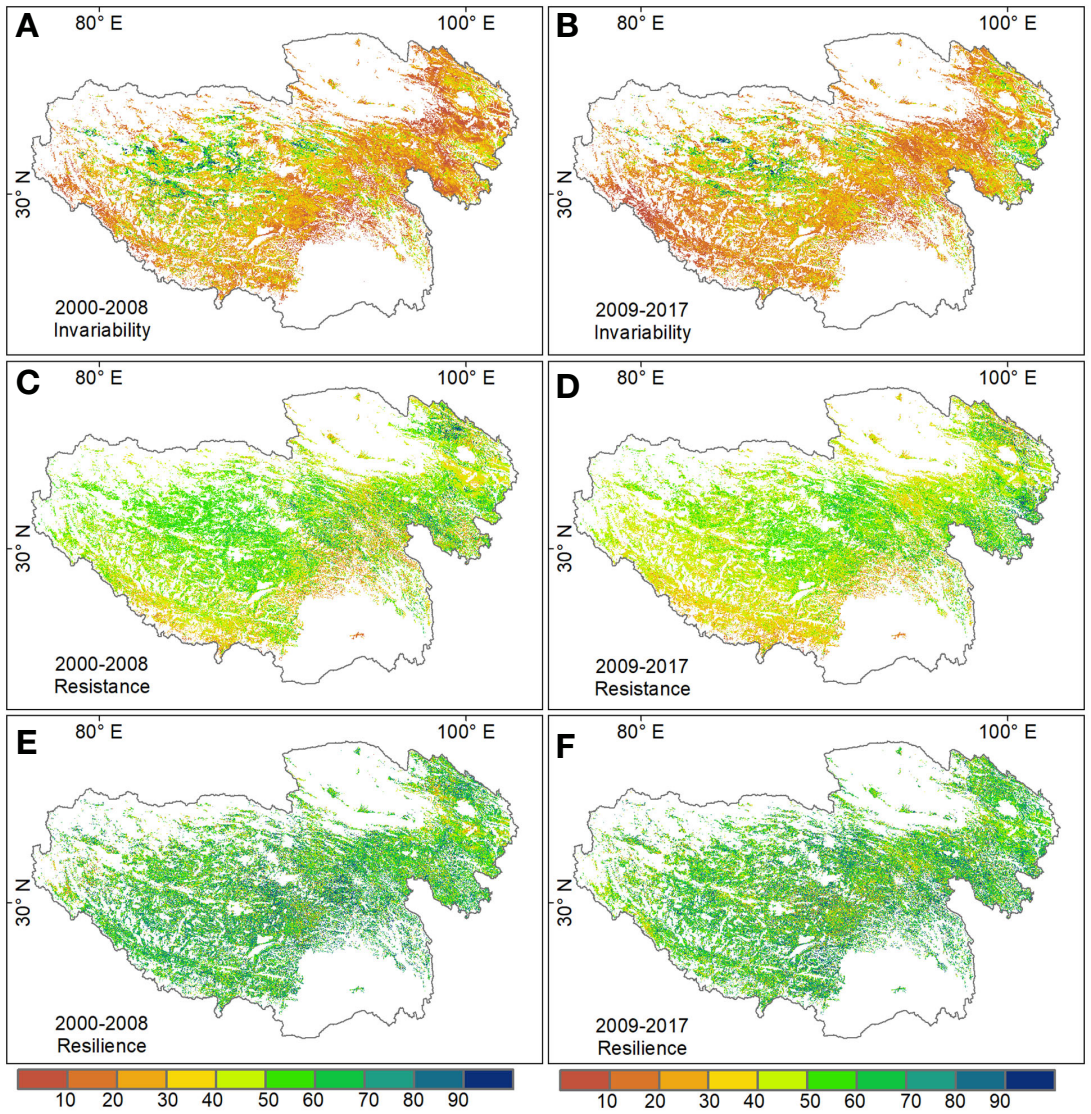
Spatial patterns of grassland standardized temporal invariability (**A** for 2000-2008, **B** for 2009-2017), resistance (**C** for 2000-2008, **D** for 2009-2017), and resilience (**E** for 2000-2008, **F** for 2009-2017) on the Tibetan Plateau.

The spatial patterns of temporal invariability, resistance and resilience of alpine grassland ecosystems were consistent in the two periods: 2000–2008 and 2009–2017 (**Figure 2**). Temporal invariability and resistance showed a clear spatial heterogeneity pattern, but the resilience showed a relative homogeneity pattern across the plateau. Especially, grasslands with high temporal invariability (> 80) occupying less than 10% of grassland areas on the Tibetan Plateau, mainly distributed in the northwest areas. The spatial pattern of resistance was partly consistent with invariability, indicating that productivity in northwestern grassland tended to be more resistant to disturbances than in other regions. Grasslands with low resilience (< 20) only accounted for less than 3% of the total grassland area, and mainly distributed in the northeast of the plateau.

Regarding different grassland types on the Tibetan Plateau, alpine steppe had the highest temporal invariability, followed by the alpine meadow and desert steppe. Resistance in alpine steppe was significantly higher than the other two grassland types in the second period (*P*< 0.05) but was similar to that in alpine meadow in the first period (*P* > 0.05). The resilience was the highest in the alpine meadow, and lowest in the desert steppe (**Table 1**).

**Table 1:** Comparisons of means ( ± standard deviation) of grassland invariability, resistance, and resilience among different grassland types on the Tibetan Plateau.

Grassland types	2000-2008			2009-2017		
	Invariability	Resistance	Resilience	Invariability	Resistance	Resilience
Tibetan Plateau	28.89 ± 17.30	46.88 ± 14.22	61.33 ± 19.57	27.31 ± 16.26	27.31 ± 16.26	60.00 ± 19.39
Alpine meadow	25.38 ± 14.20b	45.50 ± 15.10a	61.46 ± 19.78a	25.58 ± 14.20b	45.79 ± 16.70b	63.02 ± 19.54a
Alpine steppe	30.04 ± 18.17a	45.90 ± 9.50a	58.35 ± 18.80b	33.63 ± 19.79a	48.36 ± 10.02a	59.47 ± 19.36b
Desert steppe	24.60 ± 18.42c	44.28 ± 7.80b	53.16 ± 16.15c	23.28 ± 21.04c	45.78 ± 8.20b	51.4 ± 18.47c

In each column, different letters indicate significant difference between different grassland types at *P*< 0.05 level.

### Correlations among stability components and their dynamics between two periods

**Figure 3:**
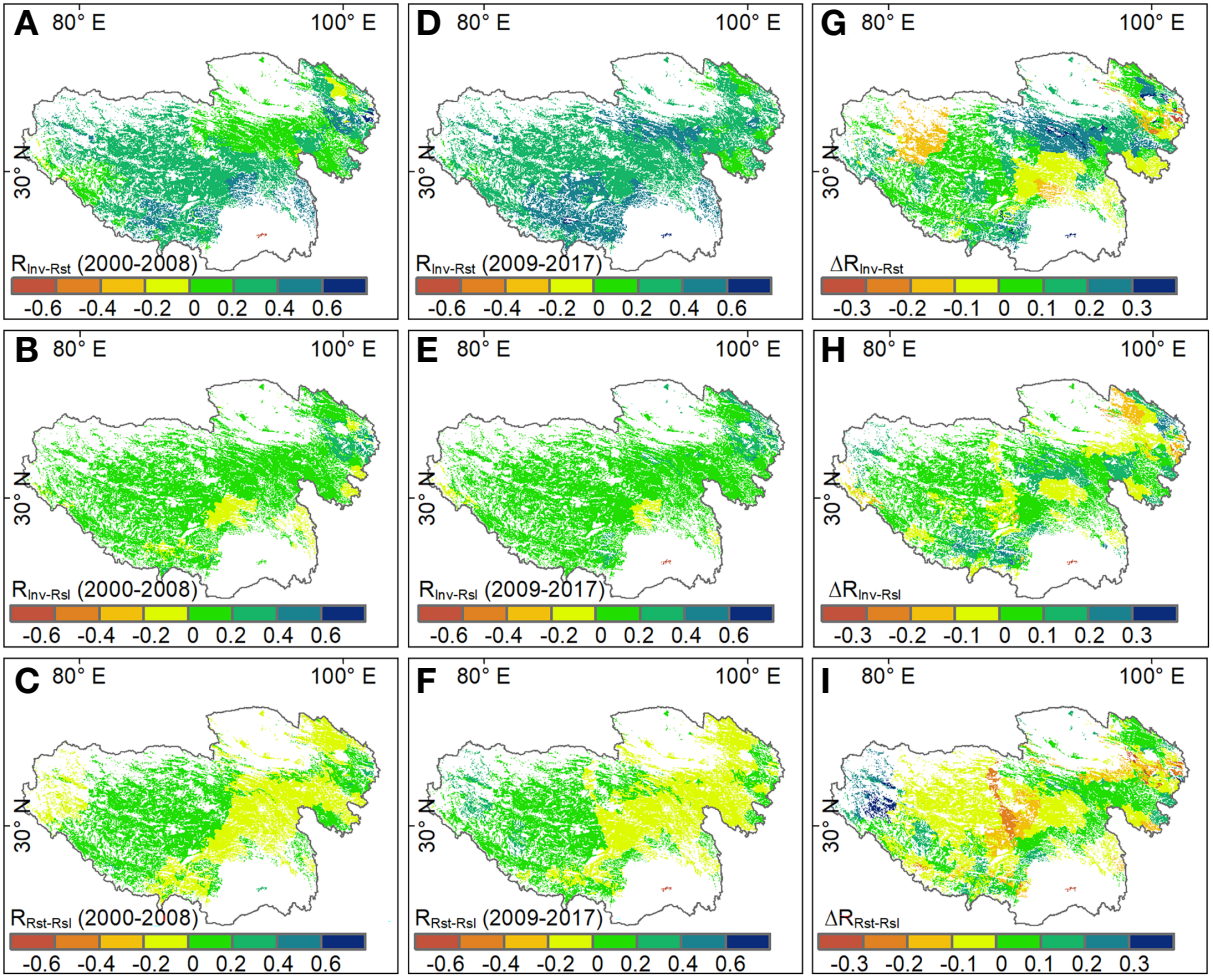
Spatial patterns of pairwise correlations among stability properties during **(A-C)** 2000-2008, **(D-F)** 2009-2017 and **(G-I)** the difference in the corresponding correlation coefficients (ΔR) between the two periods on the Tibetan Plateau. R_Inv-Rst_, R_Inv-Rsl_ and R_Rst-Rsl_ respersent the correlation coefficients between temporal invariability and resistance, temporal invariability and resilience, and resistance and resilience, respectively.

Temporal invariability and resistance showed a positive correlation for more than 90% of grassland areas on the Tibetan Plateau (R_Inv-Rst_ > 0), especially for southern areas where grasslands had a value of R_Inv-Rst_ higher than 0.4. Again, the correlation between invariability and resilience was positive (R_Inv-Rsl_ > 0) for most grassland areas. However, the correlation between resistance and resilience (R_Rst-Rsl_) showed a heterogeneous pattern, that is, R_Rst-Rsl_ was negative in eastern grasslands but positive in western grasslands (**Figure 3**).

**Figure 4:**
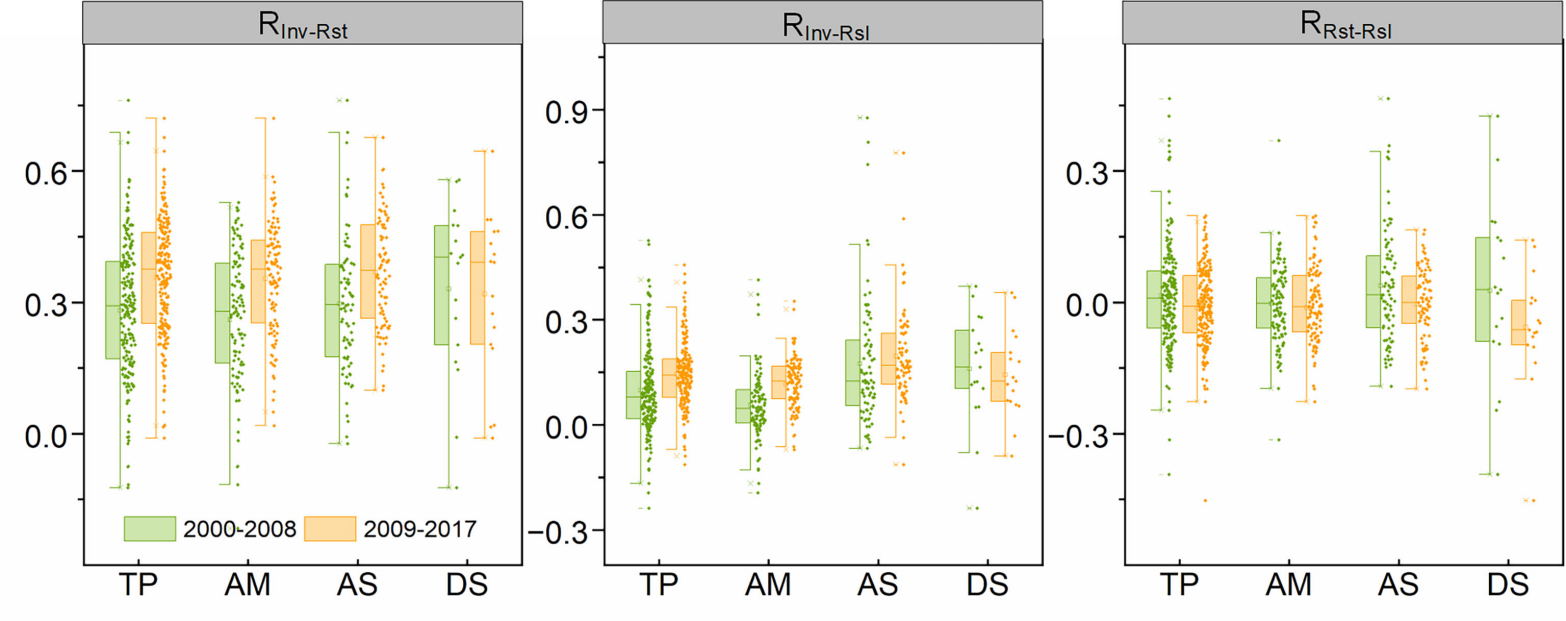
The pairwise correlation among stability properties for each grassland type in 2000-2008 and 2009-2017. TP represents the Tibetan Plateau; AM, AS and DS represent alpine meadow, alpine steppe and alpine desert steppe, respectively. R_Inv-Rst_, R_Inv-Rsl_ and R_Rst-Rsl_ represent the correlation coefficients between temporal invariability and resistance, temporal invariability and resilience, and resistance and resilience, respectively.

Although there are no significant changes in the spatial patterns of R_Inv-Rst_, R_Inv-Rsl_ and R_Rst-Rsl_ between the two periods, the magnitude of correlations among stability components has changed in the second period, compared to the first period. Especially, the average R_Inv-Rst_ and R_Inv-Rsl_ increased from 0.28 and 0.09 of the first period to 0.36 and 0.14 of the second period, respectively. However, the R_Rst-Rsl_ transformed from positive (0.01) of the first period to negative (-0.01) of the second period. For different grassland types, R_Inv-Rst_ increased in alpine meadow, alpine steppe and desert steppe, and R_Inv-Rsl_ increased in all grassland types with the exception of the desert steppe. Besides, compared with that in the first period, R_Rst-Rsl_ in all three grassland types decreased slightly in the second period (**Figure 4**).

### Dimensionality of stability

**Figure 5:**
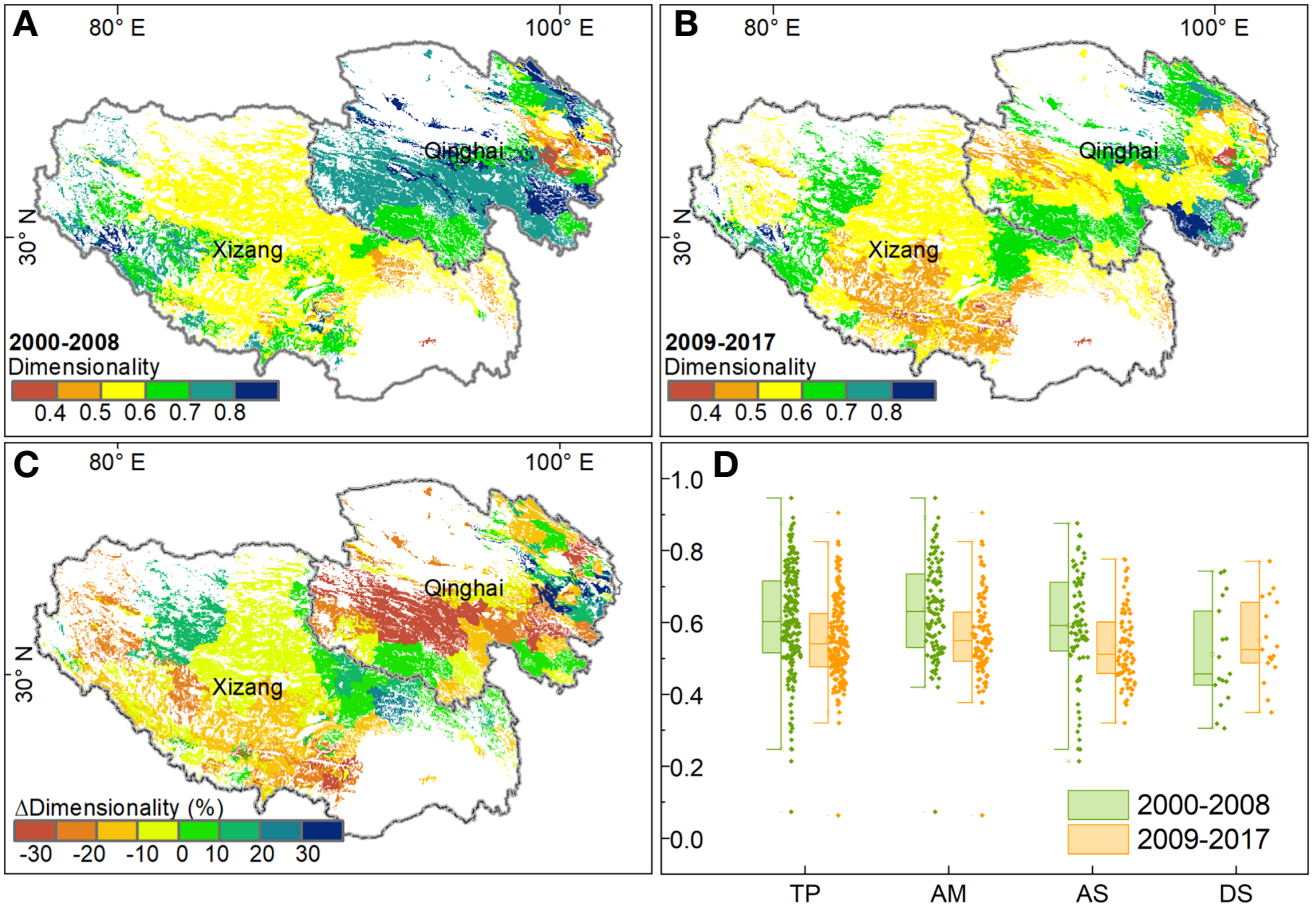
The spatial pattern of dimensionality of stability on the Tibetan Plateau: **(A, B)** dimensionality during 2000-2008 and 2009-2017, **(C)** the difference in dimensionality between the two periods, and **(D)** the boxplot of dimensionality for different grassland types.

The spatial pattern of dimensionality of stability was heterogeneous. The value of dimensionality, either in the first or second period, was high in the two sides of the Tibetan Plateau and low in the middle of this plateau (**Figures 5A, B**). For the entire plateau, the dimensionality of alpine grasslands decreased by almost 10%, from 0.61 in the first period to 0.55 in the second period. This decrease in dimensionality mostly occurred in the southwest of the Xizang Autonomous Region and the center of the Qinghai province, where the dimensionality decreased by more than 20% (**Figure 5C**). Meanwhile, we also observed a slight increase in the dimensionality in the northern Xizang Autonomous Region and northeastern Qinghai province. Statistically, the dimensionality of stability decreased in 72.7% of the study area, 26.3% of which decreased with a magnitude of more than 20%. In terms of different grassland types, the dimensionality in the alpine meadow was the highest, followed by alpine steppe and desert steppe. Comparing the two periods, we found that the dimensionality in the alpine meadow decreased from 0.64 in the first period to 0.57 in the second period. Besides, the dimensionality in the alpine steppe decreased from the first period (0.59) to the second period (0.53). In contrast, the dimensionality in desert steppe showed little increase between the two periods (**Figure 5D**).

### Relative contributions of climatic variables and grazing intensity to dimensionality

**Figure 6:**
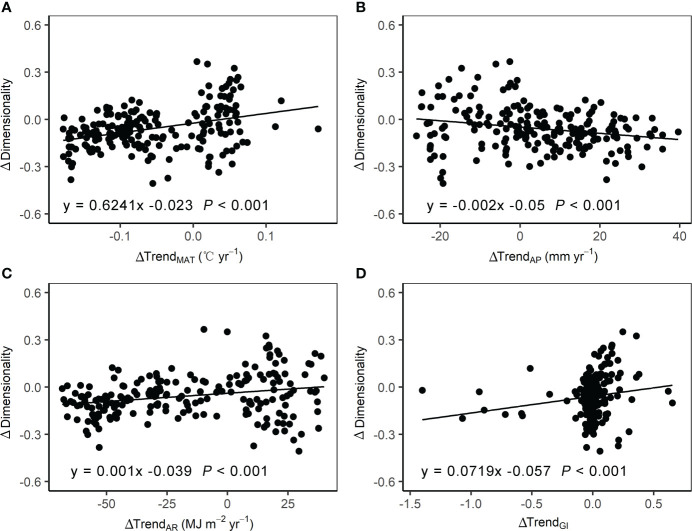
Relationships of the difference in dimensionality of stability with corresponding mean-differences in **(A)** mean annual temperature (MAT), **(B)** annual precipitation (AP), **(C)** annual radiation (AR), and **(D)** grazing intensity (GI).

**Figure 6** presents the relationships of difference in dimensionality (Δdimensionality) with differences in three climatic variables trend (ΔTrend_MAT_, ΔTrend_AP_, and ΔTrend_AR_) and grazing intensity trend (ΔTrend_GI_) between the two time periods. Δdimensionality significantly increased with increasing ΔTrend_MAT_, ΔTrend_AR_ and ΔTrend_GI_, but decreased with increasing ΔTrend_AP_.

**Figure 7:**
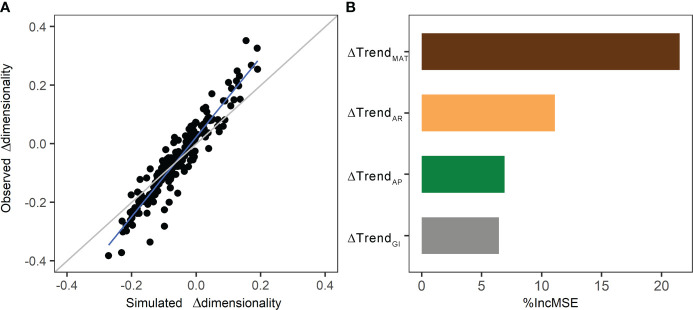
The performances of the RF model: **(A)** the relationship of observed differences dimensionality with simulated differences dimensionality during 2000–2008 and 2009–2017 (the grey line is the 1:1 line; the blue line is the regression line), and **(B)** relative contribution of predicators in RF model denoted by the percentage increase of mean squared error (%IncMSE).

RF model was conducted to measure the relative contribution of ΔTrend_MAT_, ΔTrend_AP_, and ΔTrend_AR_, and ΔTrend_GI_ to the differences of alpine grassland dimensionality. Results of the RF model show that the simulated Δdimensionality could explain 89.6% variation in observed Δdimensionality (**Figure 7A**). Among climate variables and grazing intensity, ΔTrend_MAT_ contributed the most variations of dimensionality (%IncMSE=21.5%), followed by ΔTrend_AR_ (%IncMSE=11.1%). ΔTrend_AP_ (%IncMSE=6.9%) and ΔTrend_GI_ (%IncMSE=6.4%) were the third and last important variables to Δdimensionality (**Figure 7B**).

## Discussion

### Correlations among three stability components

Temporal invariability, resistance and resilience describe different aspects of ecosystem stability in response to perturbations. Radchuk et al. (2019) suggest that the positive correlations among stability components are more beneficial to maintain a stable state of an ecosystem. For alpine grassland on the Tibetan Plateau, we find that some stability components were strongly correlated while others were uncorrelated. Especially, temporal invariability was positively correlated with resistance and resilience, while resistance was independent of resilience, indicating that ecological stability of alpine grasslands appeared to be complex. This result was in line with the findings of Donohue et al. (2013) and Radchuk et al. (2019). They suggest that one single stability component cannot capture the overall stability of an ecosystem. The high correlation between temporal invariability and resistance can be explained by the fact that both the two stability components reflect the ability of an ecosystem to maintain its initial state (Hoover et al., 2014; Zhong et al., 2019). The weak correlation between resistance and resilience did not agree with theoretical research that resistance may negatively correlate with resilience (Donohue et al., 2013), and also did not agree with Isbell et al. (2015) who find that biodiversity increase resistance but decrease resilience to climate extremes in grassland ecosystems. However, some experimental studies point out that ecosystems tend to optimize resistance-related functional traits rather than resilience-related functional traits to stabilize their functions, leading to the weak connection between resistance and resilience (Hoover et al., 2014; Baert et al., 2016). The lack of correlations between resistance and resilience suggest that it is necessary to measure the two stability components simultaneously on the Tibetan Plateau to enrich understanding of the overall ecosystem stability.

**Figure 8:**
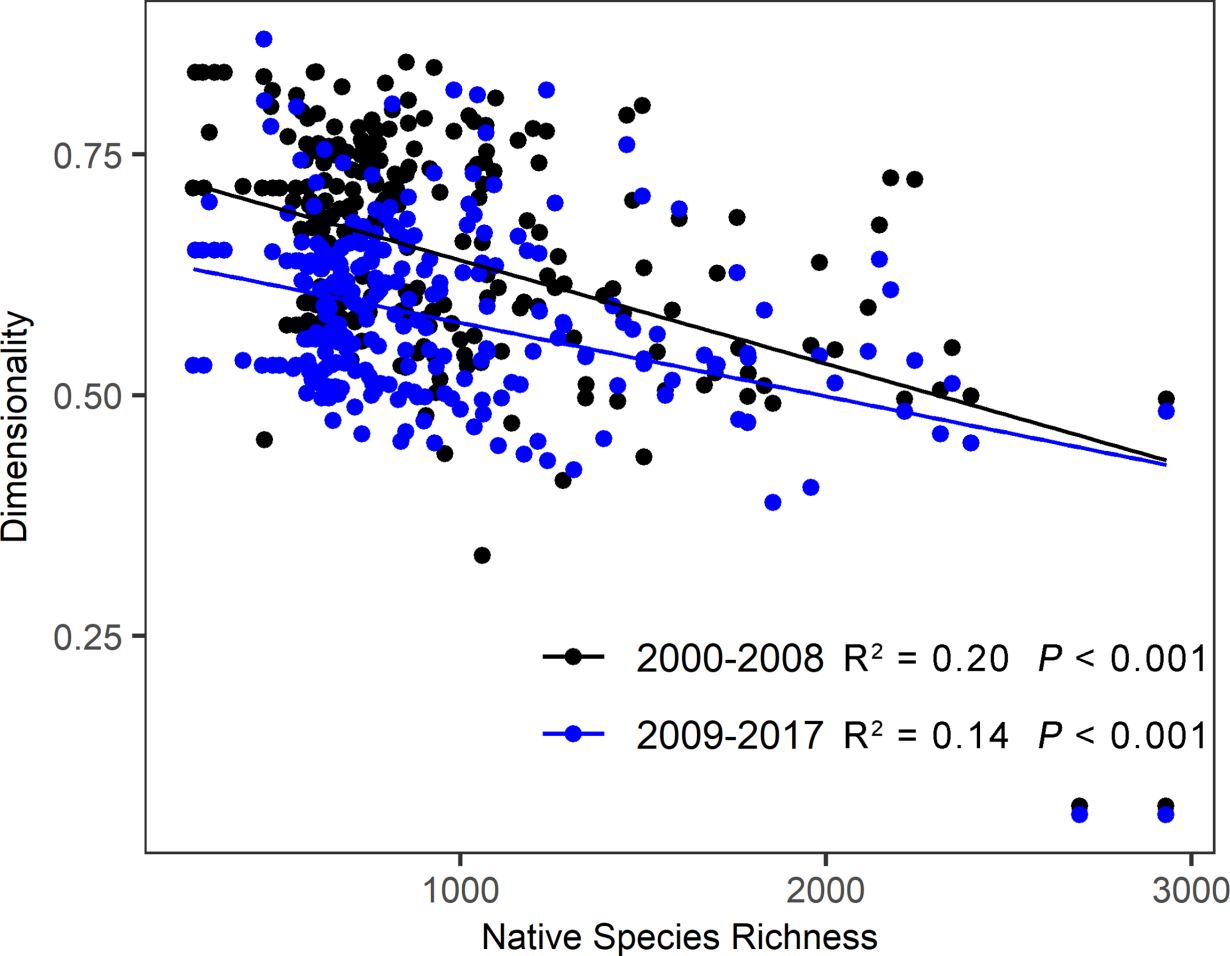
Relationships between native species richness and the dimensionality of stability at the two periods on the Tibetan Plateau. The species richness data of this plateau was extracted from the global data of vascular plant species richness (https://doi.org/10.1371/journal.pone.0030535) (Moen et al., 2012).

The dimensionality of stability captures multi aspects of stability independently. High dimensionality indicates weak connections among stability components, and *vice versa* (Donohue et al., 2013). If the dimensionality is high, policymakers should maximize one stability component without affecting other stability’s aspects (Polazzo and Rico, 2021). The research of Li et al. (2020) and Chen et al. (2021) confirm that the dimensionality of stability varies among biomes due to the differences in community characteristics and environmental conditions. In this study, we find alpine meadow had a higher dimensionality than alpine steppe and desert steppe on the Tibetan Plateau, implying that complex processes and regulatory mechanisms underlie the stability components of alpine meadow. The potential explanation is that alpine meadow has higher species richness and more complex community structure, leading to a complicated response of ecosystem stability to perturbations. According to the relationship between species richness and the dimensionality of stability, we find that alpine grasslands with higher species richness tended to shape lower dimensionality of stability (**Figure 8**). Although the large local species pool benefits grassland self-restoration on the Tibetan Plateau (Wu et al., 2017), strong competitive interactions among species usually result in weaker connection among stability components, thus generating higher dimensionality (Radchuk et al., 2019). Therefore, more stability components should be measured simultaneously to monitor and manage grassland dynamics in alpine meadows on the Tibetan Plateau.

### Effects of multiple perturbations on the relationships among stability components

Although previous studies have explored the effect of perturbations on each single stability component (Fussmann et al., 2014; Oliver et al., 2015; Ma et al., 2017), it is still not clear how perturbation affects the relationships among stability components. In this study, we show that climate variables (temperature, precipitation and radiation) and grazing have changed the magnitude of grassland stability components and their relationships. The dimensionality of stability decreased from 0.61 during 2000-2008 to 0.55 during 2009-2017, suggesting the increasing connections among temporal invariability, resistance and resilience of alpine grasslands. Previous results find that the dimensionality of stability depends on perturbation type (Radchuk et al., 2019). Hence, quantifying the relative importance of multiple perturbations on the dimensionality of stability is of great significance in diagnosing regional environmental problems and protecting critical regional ecosystems.

Both climate change and grazing activity, the major perturbations for the dynamic of grassland ecosystems on the Tibetan Plateau, significantly affected the dimensionality of stability. Although some studies disentangle the relative contributions of different perturbations to the dynamics of alpine grassland, the primary driver is still controversial. For example, Zeng et al. (2019) report that temperature has the highest contribution to the aboveground biomass variation. Zhang et al. (2016) also find that alpine grassland growth is strongly correlated with temperature. However, some studies also find that precipitation rather than warming is the primary determinant factor of plant productivity on the Tibetan Plateau (Fu et al., 2018; Yu et al., 2021). In our study, we find that temperature was the controlling factor for decreasing dimensionality on this plateau. This result is in line with the findings of a meta-analysis, in which Valencia et al. (2020) detect a significant effect of temperature on plant community stability worldwide. Climate warming on the Tibetan Plateau was faster than the global average over the past decades. However, the current air temperature is still lower than the optimum physiological temperature of leaf-level photosynthetic capacity (Huang et al., 2019). Thus, warming affects photosynthesis, community structure and species interactions of alpine grasslands significantly, and further regulates grassland stability (Zhu et al., 2016). Consequently, temperature become one of the critical explanatory climatic variables to the changes in the dimensionality of stability.

Previous studies suggest that the herbivores may decouple the relationships among temporal invariability, resistance and resilience, and thus lead to the high dimensionality of stability (Donohue et al., 2013; Chen et al., 2022). For example, Saruul et al. (2019) demonstrate that continuous grazing changes the interrelationships among stability components and further alter the dimensionality of stability. However, in our study, random forest modeling confirmed that grazing intensity explained the lowest variation of dimensionality. This finding is largely consistent with the results of Li et al. (2021a) that climate change rather than grazing activities dominate changes in grassland productivity on the Tibetan Plateau, and with the current prevailing views that climate plays a crucial role in ecosystem stability (Chen et al., 2021; Wang et al., 2022). Overgrazing or unsustainable livestock management might damage the structure and function of alpine grasslands, but such negative effects are only evident in hotspots directly adjacent to pastoralist camps (Wang et al., 2017). Thus, for the whole plateau, grazing activities might not change the dominant role of climate change for plant growth on the Tibetan Plateau.

### Suggestions for future studies

We note that this study has two limitations for further improvement. Firstly, the calculation of resistance and resilience was based on the linear relationships between NDVI and climate variables (temperature, precipitation and radiation). This method does not incorporate human activity into consideration. Since 2000, various ecological protection policies have been renewed and implemented on the Tibetan Plateau, which have driven vegetation restoration (Hua et al., 2022b). Although climate change is the dominant factor in controlling the inter-annual dynamic of grassland productivity, ecological engineering projects may have more or less impact on the changes in vegetation productivity (Li et al., 2021a; Wang et al., 2022). In this case, we might have overestimated or underestimated the contributions of climate variables to the dynamics of NDVI. Thus, in the future, we call for more complementary studies to disentangle climatic and anthropogenic contributions to the variations of vegetation productivity. Secondly, the grazing intensity was quantified at coarse spatial resolution due to insufficient data on the spatial distribution of livestock. Recently, Sun et al. (2022) have rasterized the county-scale grazing data based on the NPP data and the statistical yearbook on the Tibetan Plateau. However, this dataset only covers four years of grazing intensity, which cannot meet the requirements of trend analysis. Therefore, it is essential to produce a dataset of grazing intensity with high quality and fine spatiotemporal resolution to improve the accuracy of spatial data analysis.

## Conclusions

In this study, we use NDVI records and corresponding temperature, precipitation, and solar radiation to quantify three stability components (temporal invariability, resistance and resilience) of alpine grasslands and their relationships on the Tibetan Plateau. The findings confirm that the dimensionality of stability in the period of low human activity intensity is lower than that in the period of high human activity intensity, indicating the increasing connections among temporal invariability, resistance and resilience. Furthermore, we find that climate variability rather than grazing intensity regulates the changes in grassland stability.

## Data availability statement

The raw data supporting the conclusions of this article will be made available by the authors, without undue reservation. Requests to access the datasets should be directed to ML.

## Author contributions

ML and FH conceptualized this study. YY collected and analyzed the data. ML and YY led the writing. BN, YS, YF, and YY interpreted the results and revised the text. All authors contributed to this work and approved the final manuscript before submission.

## Funding

The study was supported by the Second Tibetan Plateau Scientific Expedition and Research (STEP) program (2019QZKK1002); the construction of the fixed Observation and Experimental Station of the first Support System for Agricultural Green Development in Zhongba County; the study on the path of agricultural green development and carbon reduction and sequestration in typical counties of Yarlung Zangbo River Basin; and National Natural Science Foundation of China (42207129).

## Conflict of interest

The authors declare that the research was conducted in the absence of any commercial or financial relationships that could be construed as a potential conflict of interest.

The handling editor declared a past collaboration with the authors ML and YS.

## Publisher’s note

All claims expressed in this article are solely those of the authors and do not necessarily represent those of their affiliated organizations, or those of the publisher, the editors and the reviewers. Any product that may be evaluated in this article, or claim that may be made by its manufacturer, is not guaranteed or endorsed by the publisher.
